# Inhibition of metastatic potential in colorectal carcinoma *in vivo* and *in vitro* using immunomodulatory drugs (IMiDs)

**DOI:** 10.1038/sj.bjc.6605206

**Published:** 2009-07-28

**Authors:** W M Liu, J Y Henry, B Meyer, J B Bartlett, A G Dalgleish, C Galustian

**Affiliations:** 1 Division of Cellular and Molecular Medicine, Department of Oncology, St George's, University of London, London, UK; 2 Celgene Corp., Summit, NJ, USA

**Keywords:** lenalidomide, pomalidomide, thalidomide, metastasis, VEGF

## Abstract

**Background:**

Thalidomide and lenalidomide are FDA approved for the treatment of multiple myeloma and, along with pomalidomide, are being investigated in various other cancers. Although these agents display immunomodulatory, anti-angiogenic and anti-apoptotic effects, little is known about their primary mode of therapeutic action in patients with cancer.

**Methods:**

As part of a continuing research effort, we have investigated the effects of these agents on the metastatic capacity of murine colorectal cancer cell lines both *in vivo* and *in vitro*. Allied to these, we have studied their effects on the molecular pathways associated with metastasis.

**Results:**

Results indicate that thalidomide, lenalidomide and pomalidomide significantly inhibit the metastatic capability of colorectal carcinoma cells. Anchorage-independent growth, used as a coarse indicator of transformation, was significantly reduced, as were migratory capacity and invasive competence. In addition, an *in vivo* experimental metastasis model also showed that treatment with the drugs resulted in a significantly lower number of metastatic pulmonary nodules relative to control mice. Allied to these cellular and phenotypic changes were alterations in molecular markers of metastasis and in intracellular signalling competency.

**Conclusions:**

These results provide evidence that in addition to their immunomodulatory effects, thalidomide, lenalidomide and pomalidomide can impair the metastatic capacity of tumours, and that this mechanism may involve alterations to cell signalling functionality.

There is growing evidence supporting a role for thalidomide-derived immunomodulatory drugs known as IMiDs in cancer therapy (Galustian *et al*, 2004; Bartlett *et al*, 2005; Shalapour *et al*, 2006; Melchert *et al*, 2007; Verhelle *et al*, 2007). This proprietary class of therapeutic analogues, as exemplified by lenalidomide (Revlimid, Celgene, Summit, NJ, USA, CC-5013) was developed on the basis of structural activity relationship studies focussing on molecular alterations to the central ring framework of the thalidomide molecule as an approach to enhancing the immunomodulatory characteristics of the agents (Stirling, 2001; Bartlett *et al*, 2004). Allied to this enhancement, IMiDs, such as thalidomide, display potent anti-proliferative, apoptotic and anti-angiogenic properties both *in vivo* and in a wide spectrum of tumour cell lines (Dredge *et al*, 2002, 2005; Shalapour *et al*, 2006; Verhelle *et al*, 2007). At present, lenalidomide is approved by the US Food and Drug Administration for use in patients with myelodysplastic syndromes (associated with no or 5q-deletions), and in combination with dexamethasone in patients with multiple myeloma who have received one previous therapy (Dimopoulos *et al*, 2007; Weber *et al*, 2007). However, there is a growing list of other cancers, including non-small-cell lung cancer, pancreatic, thymic, rectal apocrine carcinomas and angiosarcoma, in which objective responses have been observed in monotherapy or in combination with other therapies (Sharma *et al*, 2006; Kalmadi *et al*, 2007; Liu *et al*, 2009a).

Despite the clinical merit of lenalidomide, the precise mechanism of its action remains unclear, as does the primary method of action whereby clinical activity is achieved. The inhibition of metastasis, a direct anti-tumour effect, and immunomodulation have all been associated with drug action, but the importance of each appears to be dependent on the cancer type – specifically the immune status of the patient and the molecular signature of the tumour. In fact, it is possible that this broad spectrum of action actually defines its clinical activity and appeal. For example, an important facet of thalidomide and indeed the IMiDs, is the ability to inhibit metastasis and angiogenesis (D'Amato *et al*, 1994; Kumar *et al*, 2004; Chanan-Khan and Cheson, 2008). This inhibition has been attributed to a number of modes of actions, and a definitive answer is unavailable.

Angiogenesis and metastasis are hallmarks of cancer and are processes that support and propagate the disease (Folkman, 1971; Hanahan and Weinberg, 2000). They correlate with and predict tumour stage for a number of neoplasms (Weidner *et al*, 1991, 1993; Jaeger *et al*, 1995; Yuan *et al*, 1995), and as a consequence, therapeutic approaches that inhibit them have been used successfully. There are currently limited *in vitro* data exploring the anti-metastatic role of lenalidomide and pomalidomide in solid tumours, and those available have used combinations with other anti-angiogenic agents (Mangiameli *et al*, 2007; Blansfield *et al*, 2008). Therefore, the primary aim of this study was to investigate the effects of IMiDs on the metastatic capability of tumours through both *in vitro* and *in vivo* approaches. Allied to this would be to study the effects the drugs would have on intracellular signalling pathways and how they correlated with the effects on biomarkers of metastasis.

## Materials and methods

### Animals

Female BALB/c and C57BL/6 mice were purchased from and maintained by the Biological Research Facility in a pathogen-free environment at St George's (University of London, London, UK). Animals were acclimatised for at least 7 days before each experiment and were used at the age of 9–13 weeks. All procedures were conducted in accordance with and approved by the Home Office of the United Kingdom.

### Cell culture

The murine colorectal carcinoma (CRC) cell lines CT26 (syngeneic to BALB/c) and CMT93 (syngeneic to C57BL/6) were obtained from the Cancer Research UK Cell Production Laboratories (London, UK) and maintained in Dulbecco's modified Eagle's medium (DMEM; Sigma Ltd, Poole, UK) supplemented with 10% (v/v) fetal bovine serum (FBS), 2 mM L-glutamine and 1 × penicillin/streptomycin (basal growth medium). All the cell lines were incubated in a humidified atmosphere with 5% CO_2_ in air at 37 °C, and only cells with a passage number of <10 were used in the experiments.

### Reagents

Thalidomide, lenalidomide and pomalidomide were obtained from Celgene Corp. (Summit, NJ, USA), and dissolved in DMSO to create 10 mM stock solutions that were maintained at −20 °C for no longer than 1 week. For *in vivo* studies, the drugs were dissolved in 0.5% DMSO in PBS and stored at 4 °C for the duration of the experiment.

### Proliferation assays

To study the effects of thalidomide, lenalidomide and pomalidomide on cell growth, CT26 cells growing exponentially were added to 96-well plates at a density of 5 × 10^4^ per well. Drugs (1–1000 *μ*M) were then added to the wells, ensuring an equal volume of 200 *μ*l across the plates. Cell number/proliferation was measured at 72 h using a standard methylthiazoletetrazolium (MTT)-based assay without modifications. Briefly, MTT (Sigma Ltd) was added to each well to yield a working concentration of 0.4 mg ml^−1^, and the plates were returned to the incubator for a further 2 h. After this time, the medium was aspirated, 200 *μ*l of DMSO was then added to each well and the plates were agitated gently for 5 min before measuring the optical density at 540 nm in each well.

### Flow cytometric analysis of the cell cycle

The distinct phases of the cell cycle were classified by DNA staining using the fluorescent dye propidium iodide. These were measured using flow cytometry according to methods described previously (Liu *et al*, 2002). Data acquisition was performed within 1 h using a Becton Dickinson FACSCalibur cytometer (BD Biosciences, Oxford, UK), and gating was used to remove doublet artefacts and to discriminate cells from debris. A total of 10 000 cells were analysed, and the percentages of cells in G1, S and G2/M phases were determined using the cell cycle analysis program, CellQuest v3.4 (BD Biosciences).

### Colony formation in soft agar

Anchorage-independent growth, a function of transformation, was determined by assaying colony growth in soft agar. Colorectal carcinoma cells were re-suspended in DMEM supplemented with 20% (v/v) FBS and containing 0.3% (w/v) Nobel agar (Sigma Ltd), and a volume of 3 ml was poured onto a 6-well plate containing a pre-solidified agar base layer (0.5% (w/v) agar and 20% (v/v) FBS in DMEM – 2 ml). For drug treatments, cell suspensions were supplemented with thalidomide, lenalidomide or pomalidomide at 1 or 10 *μ*M. Plates were replenished every 5 days with 500 *μ*l basal growth medium (with or without drugs), and colony formation was assessed by light microscopy on day 21. The sizes of each colony were assessed, and the viabilities of each colony were confirmed by trypan blue dye exclusion.

### Cell migration and invasion assays

Experiments assessing cell migration were performed using a 24-well chemotaxis chamber consisting of a transwell permeable membrane with 3 *μ*m pore size. The chemoattractant in the lower compartment of the chamber was basal growth medium (600 *μ*l). Colorectal carcinoma cells (1 × 10^6^ ml^−1^) were re-suspended in DMEM containing 2 mM L-glutamine and 0.1% bovine serum albumin (BSA). For the treatment groups, cells were co-cultured with each drug at a range of concentrations between 0 and 10 *μ*M. Cells (300 *μ*l) were added to the upper compartment of the chemotaxis chamber, and they were allowed to traverse through the membrane for 16 h in a humidified atmosphere with 5% CO_2_ in air at 37 °C. Cells from either side of the membrane were harvested mechanically and with trypsin, before enumeration by cell counting with trypan blue dye to aid live–dead cells discrimination. The percentage of cells traversing the permeable membrane into the lower side of the chamber was then calculated.

Cell-invasive capability was assessed using a similar chemotaxis chamber set-up with few modifications. The ability of cells to invade through the basement membrane was assessed by first coating the upper surface of the polycarbonate membrane with 100 *μ*g ml^−1^ of growth factor-reduced Matrigel (BD Biosciences). Matrigel was allowed to set at 37 °C for 30 min, before adding cells to the upper compartment of the chamber. Cells were allowed to invade through the Matrigel and permeable membrane, before being harvested using the same methods described above. Cell recovery from the Matrigel layer was enhanced using the Cell Recovery Solution (BD Biosciences), and the percentage of cells in the lower compartment *vs* the upper chamber was calculated.

### Immunoblotting analysis

Cells were harvested and total cellular protein was solubilised in lysis buffer (New England Biolabs, Hitchin, UK) and resolved by Tris-glycine electrophoresis using a 4–20% gradient gel according to the method of Laemmli (1970) After transferring proteins to nitrocellulose membranes (0.45 *μ*m), blocking was performed in 5% (w/v) non-fat milk in TTBS (0.1% (v/v) Tween-20 in TBS (100 mM Tris, 150 mM NaCl, pH 7.6)). Primary antibody probing was performed with anti-phosphoAKT^ser473^, anti-AKT, anti-phosphoMAPK^erk1/2^, anti-MAPK, anti-VEGF-R1 (1 : 500 – Abcam PLC, Cambridge, UK), anti-cadherin and anti-MMP-9. All primary antibodies were obtained from New England Biolabs and used at a dilution of 1 : 1000, unless stated otherwise. Anti-GAPDH was used as a loading control (1 : 2000 – New England Biolabs). After three washing steps in TTBS, horseradish peroxidase-conjugated anti-species IgG_1_ was used as the secondary antibody (Amersham Biosciences Ltd, Little Chalfont, UK). Bands were visualised using the ECL-plus detection system (Amersham Biosciences Ltd).

### Measurement of cytokine production

CT26 cells (1 × 10^5^ cells) were cultured for 3 days in 6-well plates in the basal medium supplemented with thalidomide, lenalidomide or pomalidomide at concentrations between 0.01 and 30 *μ*M. After incubation, the media were removed, debris removed by micro-centrifugation at 13 000 *g* for 10 min and stored at −80 °C for further analysis. Changes in the expressions of a collection of metastatic-related activating and inhibiting proteins were initially screened and assessed using the TranSignal Mouse Angiogenesis Antibody Array (Panomics Inc., Redwood City, CA, USA) according to the manufacturer's protocols. Furthermore, the levels of VEGF (Peprotech EC Ltd, London, UK), IP10 and TIMP1 (both from Bender MedSystems GmbH, Vienna, Austria) were assayed using ELISA kits according to the manufacturer's instructions.

### *In vivo* murine experimental metastasis model

Exponentially growing CRC cells were harvested, washed and re-suspended in PBS at a concentration of 1.3 × 10^5^ per ml. Only cells with a viability of >90%, as established by trypan blue dye exclusion analysis, were used. Mice (BALB/c for CT26 and C57BL/6 for CMT93) were anaesthetised with isoflurane and inoculated into the tail vein with 150 *μ*l of cells. Thalidomide, lenalidomide or pomalidomide (50 mg kg^−1^) was administered daily by intraperitoneal injection. Mice were killed 14 days after tumour cell inoculation. For enumeration of pulmonary metastases of CRC cells, the lungs were perfused intra-tracheally with a 15% solution of Indian ink, resected and stabilised in Fekete's solution (70% (v/v) ethanol, 5% (v/v) formaldehyde and 750 mM glacial acetic acid in water). Metastases that appeared as white nodules on the surface of the lungs were counted independently by two operators.

A parallel experiment was also performed with CT26 cells in BALB/c mice using the slightly more active of the drugs. CT26 cells (1 × 10^5^ cell per ml) were pre-treated *in vitro* with 10 *μ*M lenalidomide for 3 days, before inoculation into mice. Mice (*n*=10) were then treated with lenalidomide or vehicle (0.5% (v/v) DMSO in PBS) for 14 days, before assessment of metastatic lesions as described above.

### Immunohistochemistry of the lungs from BALB/c mice

The lungs from BALB/c mice inoculated with CT26 cells and treated with thalidomide, pomalidomide or lenalidomide were fixed in an embedding compound and frozen in liquid nitrogen. Cryosections (6 *μ*m) were fixed onto positively charged slides in ice-cold acetone for routine haematoxylin and eosin staining (Department of Pathology, St George's Hospital, London, UK) and for assessment of VEGF-R1 levels. Slides were blocked in 5% BSA (w/v) in PBS before incubation at room temperature with anti-VEGF-R1 (1 : 50; Abcam PLC) for 3 h. After the three 5-min washing steps in PBS, Alexa Fluor 568-conjugated secondary antibody (1 : 50; Invitrogen Ltd, Paisley, UK) was applied to the slides and incubated overnight at 4 °C in darkness. The slides were washed three times in PBS and coverslips applied using mounting media containing 4′,6-diamidino-2-phenylindole (Vector Laboratories Inc., Burlingame, CA, USA). Sections were imaged using an Olympus CKX-41 inverted microscope (Olympus, East Grinstead, UK), and the VEGF-R1 expression was assessed independently by two operators.

## Results

### Thalidomide/IMiDs do not affect the proliferation of CT26 cells

**Figure 1:**
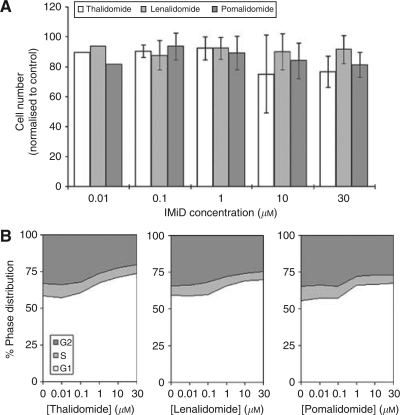
Effect of IMiDs on cell growth parameters. CT26 cells were cultured continuously with thalidomide, lenalidomide or pomalidomide (0–30 *μ*M) for 3 days. Changes in cell numbers were assessed by the MTT assay and are represented by values normalised to control samples with no treatment (**A**). The effect on cell cycle distribution was also assessed (**B**). Each data point represents the mean and s.d. (where appropriate) of at least three separate experiments.

The effect of IMiDs on cell proliferation was assayed by MTT analysis, and the concentration ranges were clinically achievable and tolerated. Results showed that thalidomide, lenalidomide and pomalidomide at concentrations ranging between 1 and 10 *μ*M had no significant effects on cell number and viability (Figure 1A). Flow cytometric analysis indicated no changes in the sub-G1 phase of the cell cycle that are suggestive of apoptosis, but highlighted a possible arrest at the G1 phase of the cell cycle and concomitant fall in the G2/M phase (Figure 1B).

### Thalidomide/IMiDs reduce the clonogenic potential of CRC cells

**Figure 2:**
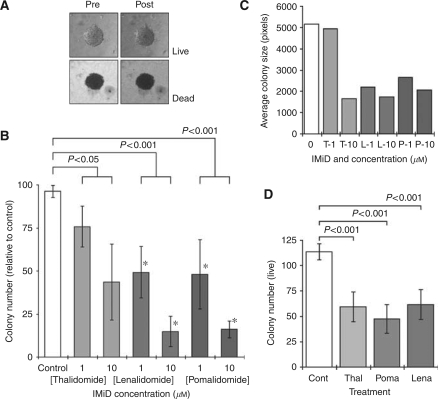
Effect of IMiDs on anchorage-independent growth. CT26 and CMT93 cells were cultured on soft agar with thalidomide, lenalidomide or pomalidomide (1 and 10 *μ*M), and colony formation assessed after 21 days. Non-viable–viable cells were discriminated using trypan blue exclusion (**A**), and the presence of live colonies in each treatment group was enumerated (**B**). ^*^P<0.05 when compared with thalidomide at the respective concentrations. The representative photographs show a live and a dead colony before and after the addition of trypan blue dye. Each column represents the mean and s.d. of three separate experiments. The average colony size was determined by measuring the area size using art software (**C**). Significant reductions in the numbers of live colonies derived from CMT93 cells were observed after culture with 10 *μ*M of any of the three drugs (**D**).

All the controls of CT26 and CMT93 cells that were treated with DMSO showed significant growth in soft agar, with an average live colony counts of 127±43 (mean±s.d.) and 114±8.1, respectively, on day 21. In CT26 experiments, the percentage of live colonies as assessed by trypan blue analysis was 96±3.5% (Figure 2A). The numbers of live colonies was significantly reduced after culturing with IMiDs (*P*<0.001) (Figure 2B). Equally, the average sizes of the colonies in the treated cultures were reduced (Figure 2C). Significant reductions in the numbers of live colonies were also observed in CMT93 cells cultured with the drugs (Figure 2D).

### Thalidomide/IMiDs reduce the FBS-mediated migration of CRC cells

**Figure 3:**
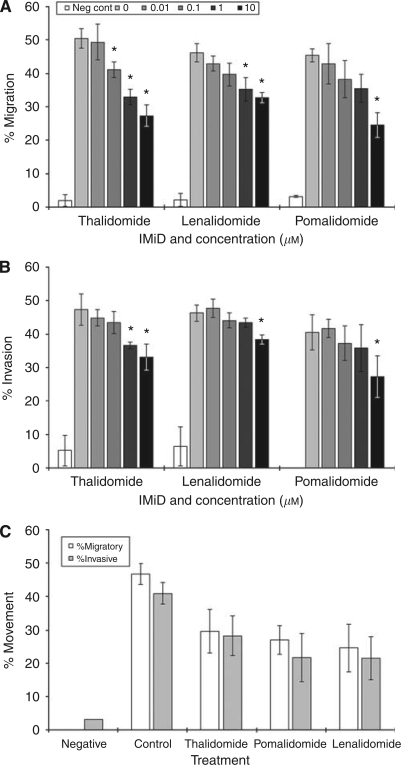
Effect of IMiDs on the migration and invasion of cells. CT26 (**A**, **B**) and CMT93 (**C**) cells were re-suspended in FBS-free growth medium in the presence of absence of thalidomide, lenalidomide or pomalidomide (0–10 *μ*M), and loaded into a chemotaxis chamber. The percentage of migratory and invasive cells was established by counting the cells in either compartment of a chemotaxis chamber after 16 h. Each column represents the mean and s.d. of at least three separate experiments. The effect of only 10 *μ*M of each drug was assessed in CMT93 (panel C).

The effect of IMiDs on the metastatic capabilities of CT26 and CMT93 cells was also examined by assessing the ability of cells to migrate across a permeable membrane towards 10% FBS in a 16 h period. (Figure 3A and C). The IMiDs were used at concentrations that were not cytotoxic at this short period of time, and the viability of cells in both compartments of the chemotaxis chambers was >90%. Migration in control wells, in which FBS was replaced with 0.1% BSA was minimal (%migration <2.4±1.5%). There was a concentration-dependent reduction in the migration of cells when cultured with IMiDs, which reach significance at the higher doses (*P*<0.001). In addition, there was no significant difference in the migratory capacity between the drugs tested.

### Thalidomide/IMiDs reduce the invasion of CRC cells through Matrigel

The movement of CT26 and CMT93 cells through a layer of extracellular matrix (Matrigel) was studied in the same chemotaxis chambers used in the migration assays. The ability of cells to invade through the layer and into 10% FBS was established by counting the number of cells located on both sides of the membrane. Cells were harvested from Matrigel using a proprietary cell recovery solution that significantly reduced cell viability; hence the percentage of invasive cells was calculated using total cell numbers rather than viable cell numbers. Results showed that IMiDs substantially reduced the percentage of invading cells (Figure 3B and C).

### Thalidomide/IMiDs alter the expression of metastatic markers

**Figure 4:**
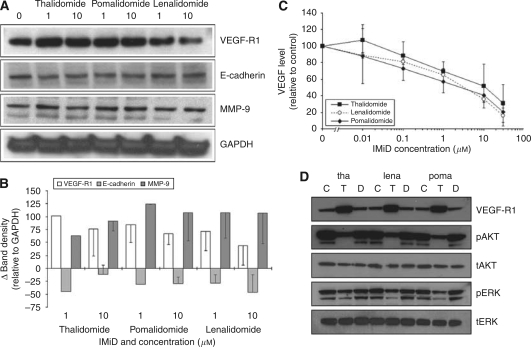
Effect of the IMiDs on the expression of metastatic markers and intracellular signalling proteins. CT26 cells were cultured with thalidomide (tha), lenalidomide (lena) or pomalidomide (poma) (0–30 *μ*M) for 3 days. The expressions of VEGF-R1, E-cadherin and MMP-9 were assessed by immunoblot analyses. Representative blots are shown (**A**), and densitometric analysis using GAPDH as the loading control was carried out (**B**). The levels of VEGF within the supernatant were also assessed by ELISA (**C**). The expressions of key signalling proteins after treatment (10 *μ*M – designated by a ‘T’) were assessed and compared with controls with no treatment (‘C’) and those with equal DMSO solvent exposure (‘D’) (**D**). Each data point is the mean and s.d. of three separate blots.

To investigate whether the changes in the macroscopic metastatic sub-systems were associated with changes in intracellular biomarkers of metastasis, whole cell lysates obtained from CT26 cells cultured with IMiDs were analysed by immunoblotting. Results showed that there was no significant effect to E-cadherin; however, there were increases in the expressions of tumour VEGF-R1 and MMP-9 (Figure 4A). Densitometry analyses of the autoradiograms showed these changes to be statistically significant compared with control samples treated with DMSO alone (Figure 4B).

### Thalidomide/IMiDs reduce VEGF production by CT26 cells

**Figure 5:**
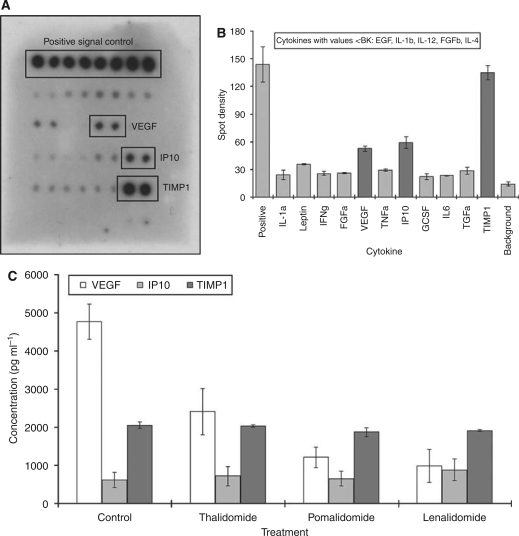
Effect of the IMiDs on the expression of angiogenic and metastatic markers. CT26 cells were cultured with the drugs for 3 days, and the growth medium was harvested. These tumour-conditioned media were subjected to assessment of proteins associated with angiogenesis and metastasis. Assessing conditioned media derived from untreated tumours indicated that some cytokines were below the magnitude background (BK) (**A**, **B**). Three cytokines, VEGF, IP10 and TIMP1, that were registered as consistently present in the media were assessed after treatment and results show that only VEGF was affected (**C**).

In an attempt to establish the cytokines and growth factors in the supernatant derived from CT26 cells, we used a proprietary sandwich ELISA kit to assess the proteins present in the medium. The signals for all the proteins on the ELISA array when assaying the basal medium (DMEM+10% FBS) were below the threshold of detection. However, a number of proteins present in the conditioned medium were flagged, of which, VEGF, IP10 and TIMP1 were consistently seen (Figure 5A and B). We next assessed the effects of the drugs on the capacity of CT26 cells to secrete these cytokines using ELISA plates. Results showed that the drugs did not affect the levels of IP10 and TIMP1; however, VEGF was significantly reduced by treatment (Figure 5C). Furthermore, the effect on VEGF was dose-dependent (Figure 4C).

### Thalidomide/IMiDs alter levels of proteins associated with intracellular signalling

To investigate whether intracellular signalling cascades were affected by treatment with IMiDs, whole cell lysates from CT26 cells cultured with 10 *μ*M of thalidomide, lenalidomide or pomalidomide for 3 days were analysed by immunoblotting. Results showed significant reductions in pAKT and pERK accompanied by an increase in VEGF-R1 levels (Figure 4D).

### Thalidomide/IMiDs inhibit pulmonary metastasis *in vivo*

**Figure 6:**
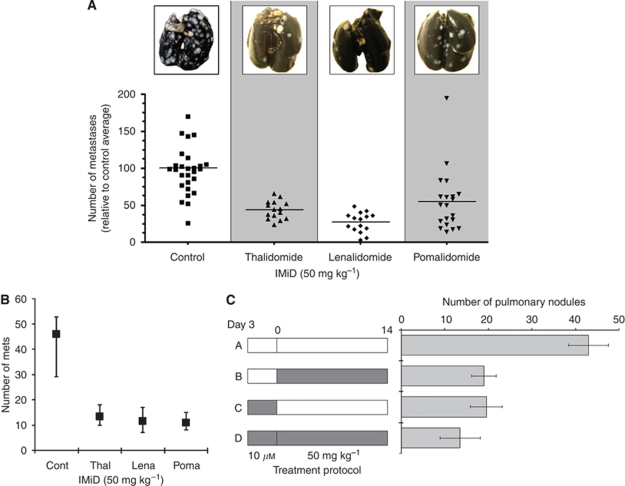
Effect of the IMiDs on pulmonary metastases. CT26 cells were injected into the tail vein of BALB/c mice. The effect of thalidomide, lenalidomide and pomalidomide administered daily IP on pulmonary metastases was assessed on day 14 (**A**). There were significantly fewer metastatic nodules in the lungs resected from treated animals (*P*<0.001 *vs* controls). Representative lungs are also shown. Similar reductions in metastatic nodules of CMT93 were observed in mice treated with the drugs (**B**). Each data point represents the means and ranges of five animals. The effect on metastasis of pre-treating CT26 cells for 3 days with 10*μ*M lenalidomide was also studied (**C**), where the shaded bars indicate treatment with lenalidomide. Pre-treatment (treatment protocol C) resulted in fewer nodules.

The propensity of CRC cells to spontaneously seed into and grow within the lungs of mice after tumour cell injection into the tail vein was used as the *in vivo* model of metastasis. The method is widely used, and easily defines metastatic potential – that is the ability of tumour cells to seed, evade host immuno-surveillance and to interact with the microenvironment by receiving growth and survival signals from it (Khanna and Hunter, 2005). Results showed that metastasis into the lung was significantly decreased in BALB/c mice treated with IMiDs compared with that in untreated mice (*P*<0.001 in all cases) (Figure 6A). As an example, the average number of pulmonary nodules from 28 untreated BALB/c mice was 95, whereas the average number in 15 mice treated daily with 50 mg kg^−1^ of thalidomide was 17. These data were recapitulated in C57BL/6 mice injected with the CMT93 cell line, with significant reductions in the number of pulmonary nodules after treatment with thalidomide, lenalidomide or pomalidomide (Figure 6B).

**Figure 7:**
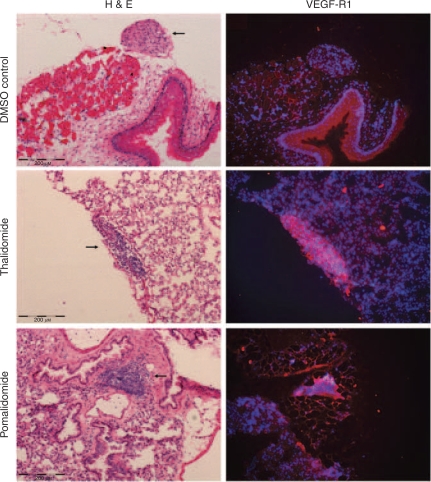
Effect of thalidomide and pomalidomide on VEGF-R1 expression in pulmonary metastases. Immunohistochemistry was performed on lung sections derived from mice treated with DMSO controls, thalidomide or pomalidomide. Metastases were identified as cellular aggregates (arrow) by H&E staining. There was an absence of infiltrating lymphocytes into these masses. VEGF-R1 expression was assessed using Alexa Fluor 568 (red) and cell nuclei counterstained with DAPI (blue), and shown to be low in the DMSO-treated group, but increased in thalidomide and pomalidomide sections. No cellular aggregates were observed in the sections from the lenalidomide-treated group.

In addition, immunohistochemistry analyses of the lungs resected from BALB/c mice showed an absence of tumour-infiltrating lymphocytes into the pulmonary cellular aggregates (Figure 7). These analyses also recapitulated the increased expression of VEGF-R1 after treatment with IMiDs compared with samples derived from the untreated control group (Figure 7).

### Pre-treatment with lenalidomide is sufficient to reduce metastasis *in vivo*

CT26 cells were pre-cultured with lenalidomide for 3 days before introduction into the mice as an attempt to understand the effect of the drugs on metastatic capability. Pre-treatment did not result in significant changes in cell number and viability. Results showed that metastasis was reduced in the untreated mice inoculated with CT26 cells pre-treated with lenalidomide (number of metastatic nodules: 19±3.7 *vs* 43±4.6 in control mice inoculated with untreated cells) (Figure 6C).

## Discussion

This study was undertaken as part of our larger remit to understand the mechanism of action of the thalidomide-related IMiDs in their inhibition of tumour progression. To this end, we specifically investigated the effects of thalidomide, lenalidomide and pomalidomide on the metastatic capability of a colon tumour cell in a number of *in vitro* and *in vivo* models. Using *in vitro* models and the drugs at physiologically achievable and clinically relevant concentrations (Galustian *et al*, 2009; Liu *et al*, 2009b), we could assess the effects on metastasis that were independent of an immune response. In addition, the colorectal CT26 cell line was used exclusively as it was syngeneic to the BALB/c mouse, which would allow *in vivo* and *in vitro* mechanistic studies to be related to each other. In summary, we confirmed that these agents significantly reduced metastasis capability directly, which was independent of a specific cytotoxic effect. Moreover, we showed that the inhibition was associated with, and mediated in part by, alterations to intracellular signalling.

The clinical value of thalidomide against solid tumours may lie in its immunomodulatory, anti-angiogenic and anti-metastatic properties. The immunomodulatory feature is believed to be a crucial determinant of clinical success and as such, analogues of thalidomide and those related to it have shown greatly increased potency for co-stimulation of T-cell and NK cells (Corral *et al*, 1999; Haslett *et al*, 2003; Aragon-Ching *et al*, 2007; Wu *et al*, 2008). These drugs have impressive activity in both haematological cancers (Galustian *et al*, 2004); however, it is not known whether the activity of solid tumours will be as strong. In haematological cancers, direct anti-survival and pro-apoptotic effects have been described for thalidomide/IMiDs that are independent of an immune response, which may be the underlying reason for therapeutic activity in neoplasia (Mitsiades *et al*, 2002; Liu *et al*, 2004). It is important to understand that the induction of tumour cytotoxicity can also be a result of an enhanced immunological responses caused by IMiDs. The increased proliferation of a number of immune cell groups, including CD4-positive and CD8-positive cells in addition to the initiation of dendritic cell function, can lead to the activation of natural killer cells that drive cytophagy and cytolysis (Davies *et al*, 2001; Hayashi *et al*, 2005; Wu *et al*, 2008).

One *in vitro* characteristic of tumour cells, which distinguishes them from normal non-tumourigenic cells, is their ability to grow competently in soft agar (Freedman and Shin, 1974; Shin *et al*, 1975). This hallmark of phenotypic transformation is observed in the majority of cancers, and occurs through the ability of the cell to gradually escape their dependence on growth regulatory mechanisms. Anchorage-independent growth has been correlated with the *in vivo* development of murine tumour cells (Cifone and Fidler, 1980) and with the propensity of tumour cells to develop metastasis *in vivo*. Our results indicated that single-cell suspension of untreated CT26 cells successfully developed into relatively large colonies of viable cells, whereas those cultured with IMiDs resulted in fewer and smaller-sized colonies, a large number of which were composed of non-viable cells. It has been shown that the gradual loss of anchorage-independent growth capability results in smaller colonies, indicating a loss of transformed phenotype (Gu *et al*, 2006). Supplementary to this, the presence of non-viable cells within these colonies suggests a cytotoxic effect (Alley *et al*, 1982); however, the concentrations of IMiDs used in the soft agar assay were not cytotoxic (Figure 1). Parenthetically, these concentrations were used in all the *in vitro* studies as they were clinically relevant and achievable in patients (Wu and Scheffler, 2004). These data suggested that the ability to initiate colony development independently of anchorage was not affected, whereas the ability to maintain growth of these colonies was compromised.

In support of the clonogenecity data, we next examined the effect of IMiDs on tumour cell motility. There is a deficit of information in this area, and although there are limited reports detailing reduced migratory characteristics in endothelial cells, there are very little data reporting these effects in tumour cells (Dredge *et al*, 2005; Komorowski *et al*, 2006). In accordance with the findings of the soft agar study, migration and invasion capacity was significantly hampered by IMiD treatment. Furthermore, the reduction in motility was not a consequence of increased cell death as the duration of exposure to drugs was 16 h and the cell viabilities in either compartment of the chemotaxis chambers were >90%. Taken together, IMiDs interfere with three key stages of metastasis – namely transformation, migration and invasion – and thus can reduce the overall metastatic capability of tumour cells. Reassuringly, the frequency of pulmonary tumours in a murine experimental metastasis model was also reduced after treatment with IMiDs, which supported the *in vitro* arm of our study. Furthermore, the reduction in pulmonary metastatic nodules in mice inoculated with tumour cells pre-treated with IMiDs, allied to the absence of lymphocyte infiltration into pulmonary cell plaques, suggested that this anti-metastatic effect of the drugs may have been independent of an immune response.

Tumourigenicity is an iterative process that requires interactions between tumour and the host microenvironment. Paracrine communications between tumour cells and endothelial cells exist to initiate, propagate and support tumour cells, and involve cross-talk between a number of chemokines, such as VEGF, IL-8 and IL-2 and their receptors (De Luca *et al*, 2008). In particular, the VEGF-R family, including VEGF-R1 and VEGF-R2, is known to be a crucial mediator of metastasis *in vivo* (Shibuya, 2001). These in turn, impact intracellular signalling cascades, such as MAPK and PI3-K (Reddy *et al*, 2003; Chan, 2004). Indeed, establishing the degree of metastasis and neovascularisation by measuring biomarkers of endothelial cell function and their interactions with tumour cells are prognostic indicators in neoplasia (Mustonen and Alitalo, 1995; Dales *et al*, 2004; Saad *et al*, 2004). Of the small group of markers studied, there was a significant increase in VEGF-R1 in CT26 cells after treatment with any of the IMiDs. This at first appeared to be counter-predicted, as these proteins are normally associated with promoting metastasis (Mustonen and Alitalo, 1995; Bergers *et al*, 2000). However, VEGF-R1 has a unique mode of action in that it has a relatively weak kinase activity even though it has a greater ligand binding affinity (Mustonen and Alitalo, 1995), and hence it functions primarily as a decoy receptor and ultimately sequesters free VEGF. Ultimately, the incidence of VEGF binding the functional subtype VEGF-R2 is reduced, which results in reduced metastatic capacity (Beck and D'Amore, 1997; Yancopoulos *et al*, 2000). Furthermore, as MMP-9 is induced by VEGF-R1, any increases in the latter would cause an increase in the former (Hiratsuka *et al*, 2002), which would explain our observations. Consequently, we explored the VEGF-R1 function further by measuring free VEGF levels and the status of signalling cascades downstream of this receptor kinase. These were reduced in all the treatment groups in which an increase in the decoy VEGF-R1 was observed. Parenthetically, as these drugs disrupt cytokine production at an mRNA level, changes in their expressions and function maybe due to post-translational processes (Liu *et al*, 2009b), which may fortify their actions.

In summary, these data reaffirm the complexity of the data surrounding the mechanism(s) of action(s) for the IMiDs, highlighting direct on-tumour effects in addition to the more classic immunomodulatory effects of this class of drug. As part of our continuing studies, we have clearly shown that the drugs significantly reduce the clonogenic and metastatic capacity of tumour cells *in vitro*. Allied to these findings, impressive reductions in the frequency of pulmonary metastases were observed in mice that appeared to be achieved independently of immune function. In addition, we have also clearly shown a reduction in cell signalling in tumour cells. Taken together, these drugs appear to reduce reverse tumourigenicity and reduce metastasis. Finally, considering the impact on VEGF-R signalling, it would be prudent to investigate the benefit of combining these agents with inhibitors of receptor tyrosine kinases; our models predicts possible enhanced effects. This is an area that we are currently pursuing, and indeed, highlights another useful facet to an already growing list of mechanisms of actions for this class of IMiDs.
